# D-dimer levels in non-COVID-19 ARDS and COVID-19 ARDS patients: A systematic review with meta-analysis

**DOI:** 10.1371/journal.pone.0277000

**Published:** 2023-02-06

**Authors:** Krisztina Tóth, Stefano Fresilli, Nicola Paoli, Giacomo Maiucci, Mariateresa Salvioni, Yuki Kotani, Stephan Katzenschlager, Markus A. Weigand, Giovanni Landoni

**Affiliations:** 1 Doctoral School of Theoretical and Translational Medicine, Semmelweis University, Budapest, Hungary; 2 Department of Anesthesia and Intensive Care, IRCCS San Raffaele Scientific Institute, Milan, Italy; 3 Vita-Salute San Raffaele University, Milan, Italy; 4 Department of Intensive Care Medicine, Kameda Medical Center, Kamogawa, Japan; 5 Department of Anesthesiology, Heidelberg University Hospital, Heidelberg, Germany; 6 University Center for ARDS and Weaning, Heidelberg University Hospital, Heidelberg, Germany; 7 Translational Lung Research Center Heidelberg (TLRC-H), Member of the German Center for Lung Research (DZL), Heidelberg, Germany; Stellenbosch University Faculty of Medicine and Health Sciences, SOUTH AFRICA

## Abstract

**Background:**

Hypercoagulability and thrombo-inflammation are the main reasons for death in COVID-19 patients. It is unclear whether there is a difference between D-dimer levels in patients without or with COVID-19 acute respiratory distress syndrome (ARDS).

**Methods:**

We searched PubMed, EMBASE, and ClinicalTrails.gov databases looking for studies reporting D-dimer levels in patients without or with COVID-19 ARDS. Secondary endpoints included length of hospital stay, and mortality data at the longest follow-up available.

**Results:**

We included 12 retrospective and 3 prospective studies with overall 2,828 patients, of whom 1,404 (49.6%) had non-COVID-19 ARDS and 1,424 had COVID-19 ARDS. D-dimer levels were not significantly higher in non-COVID-19 ARDS than in COVID-19 ARDS patients (mean 7.65 mg/L vs. mean 6.20 mg/L MD 0.88 [CI: -0.61 to 2.38] p = 0.25; I² = 85%) while the length of hospital stay was shorter (non-COVID-19 mean 37.4 days vs. COVID-19 mean 48.5 days, MD -10.92 [CI: -16.71 to -5.14] p < 0.001; I² = 44%). No difference in mortality was observed: non-COVID-19 ARDS 418/1167 (35.8%) vs. COVID-19 ARDS 467/1201 (38.8%).

**Conclusions:**

We found no difference in the mean D-dimer levels between non-COVID-19 ARDS and COVID-19 ARDS patients.

## Introduction

The COVID-19 pandemic has brought another form of acute respiratory distress syndrome (ARDS) with typical radiological findings. A severe course of the disease happens in around 5% of the cases [1]. Hypercoagulability and the risk of thrombo-inflammation are the main reasons for death [2] and lead to an impaired respiration and increased risk of pulmonary embolism [3].

Different markers have emerged to assess the risk of a severe course. D-dimer levels have been shown to be significantly elevated in patients with COVID-19 who had a severe course and subsequently died [4, 5] due to the underlying disease. D-Dimers have shown to be elevated not only in thrombosis, trauma or aortic dissection, but also in inflammation processes. On the other hand, thrombotic events were lower in patients with non-COVID-19 ARDS [6]. These findings resulted in adjusted therapeutic anticoagulant doses, which proved to be beneficial in patients with severe COVID-19 ARDS [7–9]. Therefore, monitoring D-dimer levels can be one pillar to predict the severe course and mortality in patients suffering from COVID-19 ARDS.

D-dimer levels have not been utilized to assess the risk of mortality in patients with non-COVID-19 ARDS. To this date it is unclear whether there is a difference between D-dimer levels in ARDS patients with and without COVID-19. As direct comparisons between those groups are scarce, we aimed to close this knowledge gap by conducting a systematic review with meta-analysis of studies which reported D-dimer in patients without and with COVID-19.

## Materials and methods

This study was registered at Open Science Framework Registries on July 25^th^ 2022 (Registration DOI: 10.17605/OSF.IO/PGW4K). The PRISMA checklist is provided in the S4 Table in S1 File.

Two independent researchers searched PubMed, EMBASE and ClinicalTrails.gov databases up to August 5^th^ 2022 and identified relevant articles. Disagreements were solved through consensus with the help of a senior author. The full search strategy can be found in the S1 File. Data was extracted by two investigators blinded to each other, with differences solved by consensuses.

The inclusion criteria for this study were: 1) comparison between any non-COVID-19 ARDS and COVID-19 ARDS 2) data on D-dimer level in both groups. Exclusion criteria were 1) patient population included non-ARDS patients 2) no available data about D-dimer level. We did not apply age or language restriction.

The primary end point was to compare the level of D-dimer levels between groups. Furthermore, we considered C-reactive protein (CRP) levels, fibrinogen levels, PaO2/FiO2 ratio, length of intensive care unit (ICU) stay, length of hospital stay, mortality data at the longest follow-up available as secondary end points. In cases where D-dimer levels were reported more than once we used the baseline data.

### Risk of bias

We used the Newcastle-Ottawa Quality Assessment Scale (NOS) for cohorts and case control studies which contains 8 items within 3 domains. The domains ‘Selection’ and ‘Outcome’ contain four and three items, respectively. Each item can be awarded one point. For the domain ‘Comparability’ up to two points can be awarded. This results in a maximum score of 9. A study was considered of high quality if it scored 7 to 9 points; fair quality for 4 to 6 points and poor quality for 0–3 [10]. Risk of bias was investigated by two authors independently. Any disagreement was solved by consulting a senior author or through consensus. For the primary endpoint visual inspection of the funnel plot was used to assess the publication bias and a possible small study effect.

### Statistical analysis

To analyze data we used Review Manager (RevMan) Version 5.4.1 (Review Manager, The Nordic Cochrane Centre, The Cochrane Collaboration, Copenhagen, Denmark) and STATA 13.0 (Stata Corporation, College Station, TX). We used Cochran’s *Q* test and *I*^2^ statistic to assess heterogeneity of the results. The value of *I*^2^ was considered ≥50%—high statistical heterogeneity, in this case random-effect model was used. If the heterogeneity was low fixed effect was used. Mantel-Haenszel statistical method was used to calculate pooled mean difference (MD) or standardized mean difference (SMD) with corresponding 95% confidence intervals (CI) in cases of continuous outcomes, while for dichotomous variables odds ratios (ORs) or risk ratio (RR) were used with 95% (CI). When the continuous variables were given as medians and IQRs we used the method proposed by Wan et. al [11] to concert data means and SDs. If the p value was less than 0.05, it was considered statistically significant. In cases were the laboratory parameters expressed in different units, they were brought to a common denominator. When no numerical data were available, we used the PlotDigitizer v3. 2022 free version to extract data form graphs. Two articles gave D-dimer data without units of measurement, in these cases we reached out to the authors to give us the precise units of measurement. In one case the authors replied and gave us the required data. Because we did not receive an answer from the authors of the other article, we used the ones employed in recent papers reporting D-dimer levels published by the same author and institution, assuming consistency for methodology of laboratory assessment.

### Sensitivity analysis

After data extraction we defined two sensitivity analysis for the primary outcome. First, we excluded studies that did not achieve ≥7 points on the NOS scale. Secondly, as D-dimer units were not available for one of the studies, units from prior publications of the respective author was assumed. To assess any risk of bias introduced we performed sensitivity analyses excluding the respective studies for the primary outcome. We compare the result of the sensitivity analysis against the original primary outcome result.

## Results

**Fig 1:**
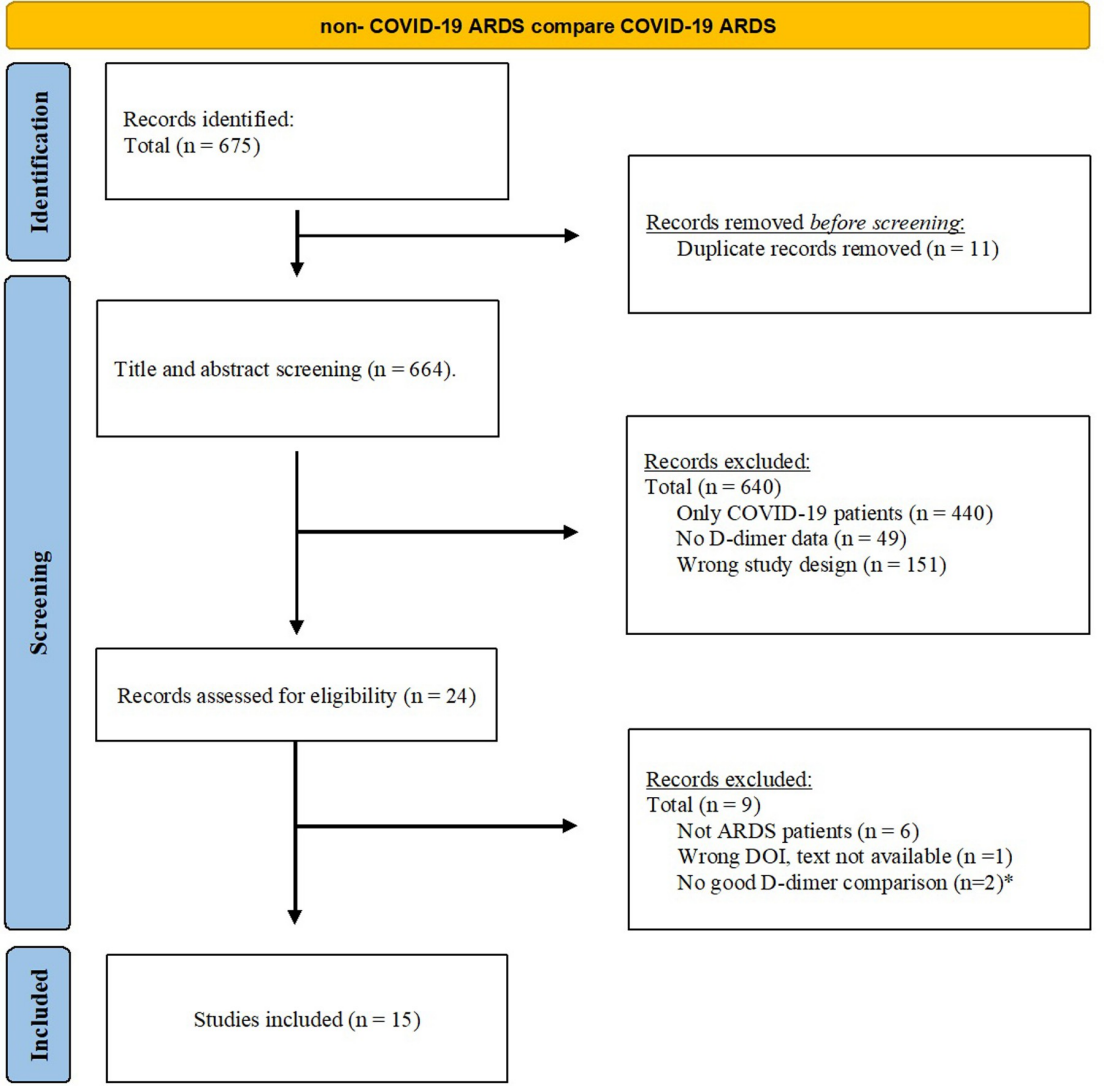
Flow chart. No good D-dimer comparison: In one case (G. Grasselli) they only reported D-dimer in COVID patients and in the other case (P. Sinha) there were no non-COVID comparison.

After removing duplicates, two independent researchers screened the titles and abstracts of 675 items and 24 articles were found to be eligible for full text reviewing. After excluding case reports, study protocol and studies not reporting primary data, we included 15 studies [12–26] in the final analysis (Fig 1).

**Fig 2:**
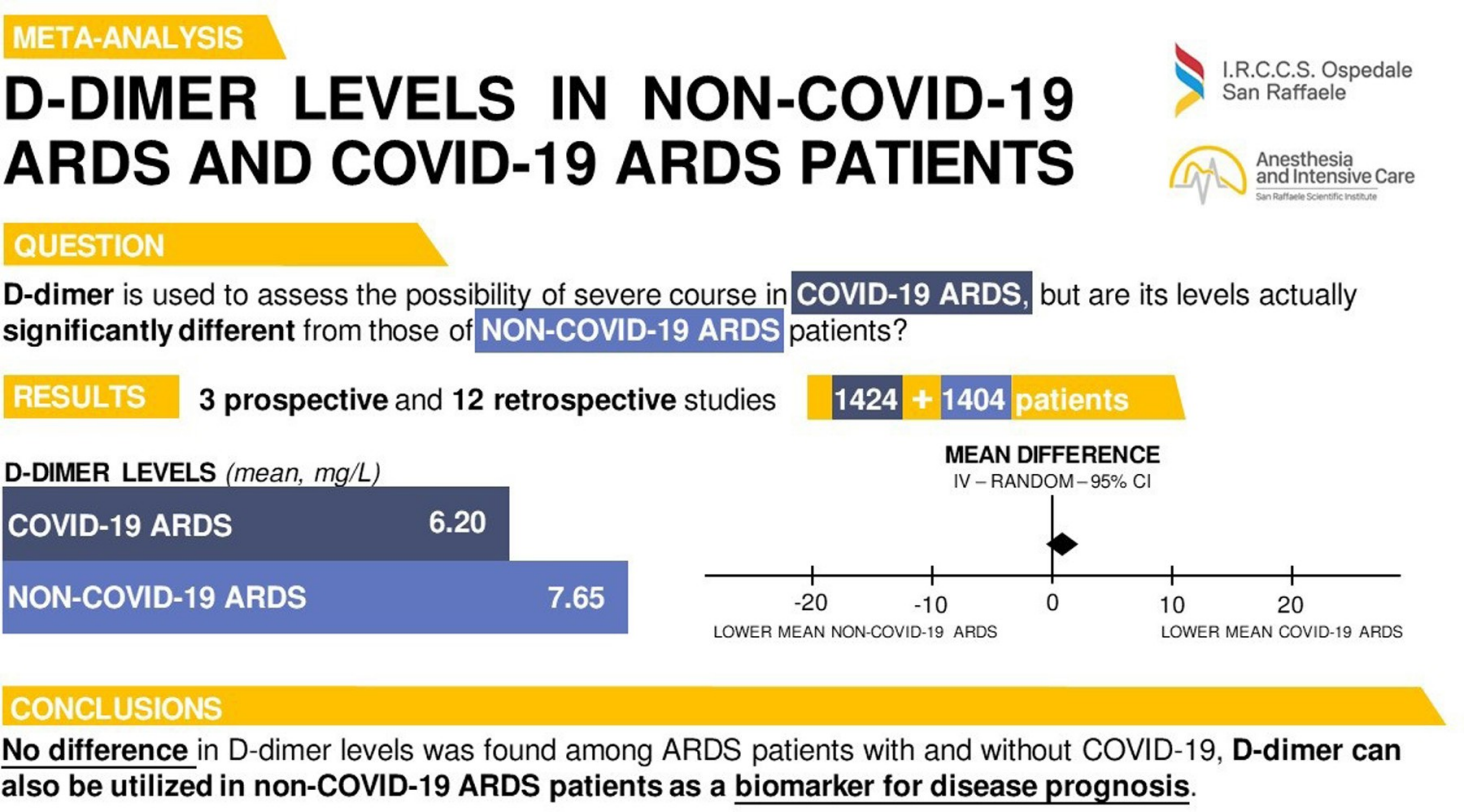
D-dimer levels in non-COVID-19 ARDS and COVID-19 ARDS patients. Visual summary of the main steps of our research.

Studies included in the present manuscript were conducted in 6 countries (USA, UK, Germany, France, Italy, China). Most studies (n = 12) were retrospective [12–17, 20–24, 26], and the only 3 prospective studies included historical cohorts of non-COVID-19 ARDS patients [18, 19, 25]. In total, data from 2,828 patients were included in our meta-analysis, with 1,404 (49.6%) having non-COVID-19 ARDS and 1,424 (50.4%) COVID-19 ARDS (Fig 2). The characteristics of the studies, including the timepoint of D-dimer sampling, are shown in Table 1.

**Table 1:** Characteristics of included studies.

First author	Publication date	Study design	Country	Multicentre	non-COVID ARDS comparator	Number of COVID ARDS patients	Number of non-COVID ARDS patients	All patients on ECMO	Longest follow-up available	D-dimer data collection time
T. Autschbach [26]	2021	observational, cohort, propensity matched	Germany	no	Influenza A (H1A1)	19	34	yes	90 days	before ECMO
A. J. Doyle [15]	2021	retrospective	UK	no	Influenza	51	80	yes	-	before ECMO
V. Fanelli [17]	2022	retrospective	Italy	yes	H1N1	146	162	yes	90 days	before ECMO
G. Hékimian [18]	2021	prospective, cohort	France	no	severe influenza pneumonia, bacterial pneumonia, sepsis	20	10	yes	on ECMO	before ECMO
J. Helms [19]	2020	prospective and historical prospective cohort	France	yes	bacterial, viral	150	233	no	7 days	at ICU admission
D.J. Hoechter [20]	2020	retrospective	Germany	no	any kind	22	14	no	-	within 48 hours from ICU admission
C. N. Lang [22]	2021	retrospective, observational	Germany	no	any kind	47	116	not all (COVID-ARDS 14/47 vs. non-COVID ARDS 49/116)	ICU stay	at admission
M. Lemzey [21]	2020	retrospective, observational, case-control	France	no	any kind	44	39	no	28 days	at ICU admission
K. A. Northam [24]	2022	retrospective, cohort	USA	no	influenza	31	14	yes	in hospital mortality	during ECMO
L. A. Raff [12]	2022	retrospective	USA	no	influenza	32	28	yes	60 days	before ECMO
B. Seelinger [23]	2022	retrospective	Germany	yes	any kind	142	68	yes	-	before ECMO
M. W.Sjoding [14]	2021	retrospective, cohort	USA	no	any kind	130	382	no	28 days	within 48 hours from intubation
S.Spadaro [25]	2021	prospective	Italy	no	any kind	31	10	no	-	within 48 hours from start of mechanical ventilation
S. Yin [16]	2021	retrospective	China	no	any kind	449	104	no	28 days	at the time the patients met the definition of severe illness
J. Zhang [13]	2021	retrospective	China	yes	non-viral	90	130	no	-	at ICU admission

ARDS = acute respiratory distress syndrome; ECMO = extracorporeal membrane oxygenation; ICU = intensive care unit.

### Risk of bias assessment

Based on the Newcastle-Ottawa Quality Assessment Scale one article was considered fair quality [14] since more than 50% of D-dimer data were missing. All the others were considered high quality (S1 Table in S1 File).

#### Meta-analysis

There was no significant difference between the mean ages in the two groups with a MD of -1.74 year (non-COVID-19 ARDS 55.4 years vs. COVID-19 ARDS 57.7 years) (95% CI: -5.14 to 1.66; I² = 87% p = 0.30). The pooled non-COVID-19 ARDS group had 910/1383 (66%) males while the COVID-19 ARDS group included 925/1331 (69%) males (p = 0.36).

**Fig 3:**
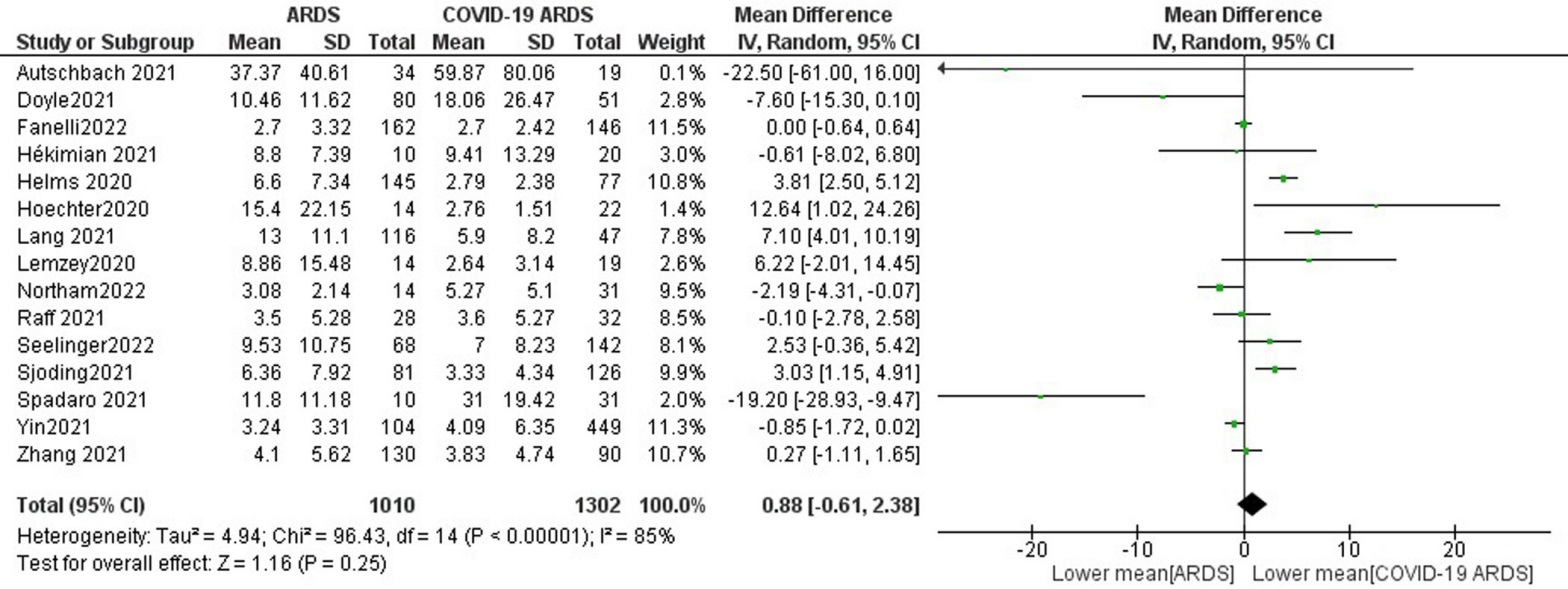
Forest plot of D-dimer level. A comparison of mean D-dimer levels between non-COVID-19 patients [ARDS] and COVID-19 patients [COVID-19 ARDS]. There was no statistically significant difference between the mean D-dimer levels in the two groups (p = 0.25).

D-dimer levels were not significantly increased in non-COVID-19 ARDS patients compared to COVID-19 ARDS with pooled mean values of 7.65 mg/L and 6.20 mg/L, resulting in a MD of 0.88 mg/L (95% CI -0.61 to 2.38, p = 0.25) (Fig 3) with sensitivity analyses confirming these findings when removing the study with unclear units of measurement (S11 Fig in S1 File) or when excluding the study with <7 points on the NOS scale (S9 Fig in S1 File). In a subgroup analysis, no difference was confirmed for ECMO patients (8 studies): -3.20 mg/L (95% CI: -5.72 to -0.68, p = 0.33) (S1.2.2 Fig in S1 File).

The non-COVID-19 ARDS patients had statistically lower fibrinogen levels (mean 454 mg/dl vs. 653 mg/dl) [12–15, 18–21, 25, 26] when compared to COVID-19 ARDS patients resulting in a MD of -173 mg/dl (95% CI: -232.48 to -113.63) p<0.001 (S3 Fig in S1 File). In contrast, CRP levels [13–15, 18, 20–23] (MD -1.07 mg/dl [95% CI: -5.08 to 2.95, p = 0.60]) and PaO2/FiO2 ratio [12, 13, 17–25] (MD -2.75 mmHg [95% CI: -10.15 to 4.65, p = 0.47]) were similar between groups (S2 and S4 Figs in S1 File).

When assessing length of ICU stay, we found no difference between non-COVID-19 (mean 21.4 days) and COVID-19 patients (mean 31.9 days) [13, 17, 22] with a MD of -6.18 days (95% CI: -24.11 to 11.75, p = 0.50) (S5 Fig in S1 File). On the other hand, time on ECMO (non-COVID-19 mean 16.0 days vs. COVID-19 mean 17.8 days) [12, 15, 17, 19, 22–24, 26] and length of hospital stay (non-COVID-19 mean 37.4 days vs. COVID-19 mean 48.5 days) [17, 24, 26] were both significantly shorter in non-COVID-19 ARDS patients with a MD of -3.20 days (95% CI: -5.72 to -0.68, p = 0.01) (S8 Fig in S1 File) and -10.92 days (95% CI: -16.71 to -5.14, p<0.001) (S6 Fig in S1 File), respectively.

No significant difference was noted in mortality (non-COVID-19 ARDS 418/1167 (35.8%) vs. COVID-19 ARDS 467/1201 (38.8%)) [12–18, 21–24, 26] (S7 Fig in S1 File) at the longest follow-up available with an OR of 0.75 (95% CI: 0.49 to 1.16, p = 0.20).

## Discussion

In this systematic review and meta-analysis, we found no significant differences between D-dimer levels in patients with non-COVID-19 ARDS compared to those with COVID-19 ARDS. Furthermore, in contrast to previously published data [6] we found no difference in mortality between the two groups. On the other hand, we found statistically significant higher fibrinogen levels, longer length of hospital stay and time on ECMO in COVID-19 ARDS patients.

Increased D-dimer levels have been shown to be a good predictive biomarker for severe course of COVID-19 at admission [27, 28]. While prior studies [27, 29] suggested to use a cut-off value of 1.5mg/L or 1.8mg/L in COVID-19 patients, we found distinct higher pooled mean values for D-dimer for both groups at admission with 7.65 mg/L and 6.20 mg/L for non-COVID-19 and COVID-19 ARDS patients, respectively. In addition, mortality rate was higher in our studies (35.8% in non-COVID-19 ARDS and 38.8% in COVID-19 ARDS patients) compared to 18.7% by Poudel et al. A difference in mortality can be explained by the exclusion criteria in the study by Poudel et al. as patients with deep vein thrombosis and/or pulmonary embolism were excluded.

D-dimer in our meta-analysis was higher than the values found in a previous systematic review by Rostami et al [30] where mean D-dimer level was 3.55μg/ml in patients with a severe course of COVID-19. Furthermore, increased D-dimer levels on admission were associated with a higher risk of mortality in a meta-analysis by Gungor et al [31]. This is corroborated by Bansal et al [32], adding that also risk of ICU admission and ARDS were associated with elevated D-dimer levels [32].

Due to the limited data, we were not able to perform a meta-regression, assessing a cut-off point for D-dimer to predict mortality in our study. While the optimal cut-off point for predicting mortality in COVID-19 patients remains unclear due to the heterogeneity of the disease, values vary between 0.67 to 2.025 μg/ml with a large variance in accuracy [33–36].

In our focused systematic review with meta-analysis, we corroborated the importance of D-dimer levels for patients without and with COVID-19 ARDS. Since patients with severe ARDS are scarce, we add a considerable value to the current literature by our analysis. Taking into account that we found no difference for D-dimer levels between non-COVID-19 ARDS and COVID-19 ARDS, we suggest that D-dimer can be utilized also in non-COVID-19 ARDS patients as a biomarker for disease prognosis. Our study found substantially increased mean D-dimer values for non-COVID 19 ARDS patients with a high case fatality rate, raising the question what an ideal cut-off point for the prediction of mortality in this setting can be. Other systematic reviews focused solely on COVID-19 ARDS, leaving a gap in the literature. With this work we try to close the knowledge gap of differences in D-dimer levels between non-COVID-19 ARDS and COVID-19 ARDS patients. In our study non-COVID-19 ARDS patients had lower fibrinogen levels, shorter length of hospital stay and shorter time on ECMO. However, this did not result into an overall survival benefit, compared to COVID-19 ARDS patients.

Our sensitivity analysis found no substantial risk of bias introduced by the study with a medium quality rating in the NOS. Overall MD in D-dimer levels was 0.88 mg/L (95% CI -0.61 to 2.38), compared with MD 0.97 mg/L (95% CI -0.62 to 2.56) in our sensitivity analysis. Furthermore, we excluded one study were D-dimer unit was assumed based on prior publications by the respective author. Again, MD did not differ compared to our primary analysis with 0.65 mg/L (95% CI -0.92 to 2.22). Overlapping confidence intervals suggested no significant differences in MD of D-dimer levels.

The strengths of our study lie within the rigorous methods in accordance with PRISMA guidelines and risk of bias assessment according to the Newcastle Ottawa Scale. We performed our search accounting for the latest publications with a broad geographic distribution. To our knowledge, this is the first systematic review with meta-analysis that highlights the differences in D-dimer levels between non-COVID-19 ARDS and COVID-19 ARDS patients. We were able to perform subgroup analysis for patients requiring ECMO support and analysed additional factors which lead to a better comparison between those two groups. To assess the possibility of risk of bias we performed two sensitivity analyses for the primary outcome.

On the other hand, our findings are limited by: 1 the lack of prospective matched cohort studies; 2 high heterogeneity between the studies across all subgroups; 3 the fact that anticoagulation treatment was not sufficiently reported in the respective studies and could therefore not be analyzed; 4 most non-COVID19 patients in this study were patients with influenza, thus generalizing these results to other (non-influenza) infective and non-infective causes of ARDS may not be appropriate.

In addition, this work highlights the lack of cohort studies and calls for future studies to assess the influence of elevated D-dimer levels on the therapy in both non-COVID-19 and COVID-19 ARDS patients.

## Conclusion

In our meta-analysis we found no significant difference in mean D-dimer levels between non-COVID-19 ARDS and COVID-19 ARDS patients. Although length of hospital stay and time on ECMO were significantly shorter in non-COVID-19 ARDS, both groups had a high case fatality rate of over 30%.

## Supporting information

S1 File Contains supporting materials, tables, figures and references. (DOCX)
