# Supplementary material for: D-dimer levels in non-COVID-19 ARDS and COVID-19 ARDS patients: A systematic review with meta-analysis
Source: PLoS One. 2023 Feb 6;18(2):e0277000. doi: 10.1371/journal.pone.0277000 (PMC9901787; doi:10.1371/journal.pone.0277000)
Supplement: S1 File — (DOCX) [file pone.0277000.s001.docx]

**SUPPLEMENTARY MATERIAL**

**D-dimer levels in non-COVID-19 ARDS and COVID-19 ARDS patients: a meta-analysis**

**INDEX** P1

**SUPPLEMENTARY MATERIAL 1** Search string P2

**SUPPLEMENTAL TABLE 1** Risk of Bias- NEWCASTLE – OTTAWA QUALITY ASSESSMENT SCALE P3

**SUPPLEMENTAL TABLE 2** Characteristics of excluded studies P4

**SUPPLEMENTAL FIGURE 1** Forest plot of D-dimer levels in subgroups P5

**SUPPLEMENTAL FIGURE 2** Forest plot of CRP levels P6

**SUPPLEMENTAL FIGURE 3** Forest plot of fibrinogen levels P7

**SUPPLEMENTAL FIGURE 4** Forest plot of PaO2/FiO2 ratio P8

**SUPPLEMENTAL FIGURE 5** Forest plot of length of Intensive Care Unit stay P9

**SUPPLEMENTAL FIGURE 6** Forest plot of length of hospital stay P10

**SUPPLEMENTAL FIGURE 7** Forest plot of mortality data at the longest follow-up available P11

**SUPPLEMENTAL FIGURE 8** Forest plot of ECMO duration P12

**SUPPLEMENTAL FIGURE 9** Forest plot of D-dimer levels (sensitive analysis)

P13

**SUPPLEMENTAL FIGURE 10** Funnel plot of D-dimer levels P14

**SUPPLEMENTAL FIGURE 11** Forest plot of D-dimer levels (removed unclear units) P15

**SUPPLEMENTAL TABLE 3** Thrombotic events (non-COVID ARDS vs. COVID ARDS) n/N”. P16

**SUPPLEMENTAL REFERENCES**  P17

**Supplemental Material 1 - Search string**

((Ventilated [tiab] or Sepsis* [mh] or Extracorporeal Membrane Oxygenation [tiab] or ECMO [tiab] or ICU [tiab] or intensive care [tiab] or critical care [tiab]) and (COVID-19 [tiab] or covid- 19 [mh] or SARS-CoV-2 [mh] or SARS-CoV-2 [tiab] or ncov-19 [tiab]) and (non-SARS-CoV2 [tiab] or Other Viral [tiab] or Acute Respiratory Distress Syndrome [tiab] or Systemic Inflammatory Response Syndrome [Mesh] OR "Bacteremia"[Mesh] OR "Sepsis"[Mesh] OR "Shock, Septic"[Mesh] OR sepsis[tiab] OR bacteraemia*[tiab] OR bacteremia*[tiab] OR septic[tiab] OR systemic inflammatory response syndrome[tiab] OR SIRS[tiab] OR septicemia*[tiab] or influenza [tiab] or Respiratory Distress Syndrome* [mh] or bacterial pneumonia [tiab]) and (dimer [tiab] OR D-dimer [tiab] or coagulation [tiab] or hypercoagulable [tiab] or coagulopathies [tiab] or Respiratory Distress Syndrome* [mh])NOT (meta-analysis[pt] OR practice-guideline[pt] OR review[pt] or Systematic Review [ti] or study design [ti] or editorial [pt] or Pediatric [ti] or vaccination* [ti] or case reports [pt] or children [ti] or vaccine* [ti] or survey [ti] or comment [pt] or case report [ti]))

**Supplemental Table 1 - Risk of Bias- NEWCASTLE - OTTAWA QUALITY ASSESSMENT SCALE**

| **First author** | **Score** |
| --- | --- |
| Autschbach | 7 |
| Doyle | 7 |
| Fanelli | 7 |
| Hékimian | 7 |
| Helms | 7 |
| Hoechter | 7 |
| Lang | 7 |
| Lemzey | 7 |
| Northam | 8 |
| Raff | 7 |
| Seelinger | 7 |
| Sjoding | 6 |
| Spadaro | 7 |
| Yin | 8 |
| Zhang | 7 |

A scale for the assessment of the quality of nonrandomized studies in meta-analyses. It contains 8 items within 3 domain and the total score is 9. A study with score from 7-9 has high quality, 4-6 fair quality, and 0-3 high risk of bias [1]

**Supplemental Table 2 – Characteristics of excluded studies with reason for exclusion**

| **First author** | **Publication date** | **Study design** | **Country** | **Multicentre** | **non-COVID ARDS type** | **Reason for exclusion** |
| --- | --- | --- | --- | --- | --- | --- |
| G. Grasselli[2] | 2020 | prospective, observational | Italy | yes | not specified | no good D-dimer comparison |
| Y. Mei[3] | 2020 | retrospecitve | United States | no | Influenza | not ARDS* population |
| D. Papadakis[4] | 2022 | prospective, observational | Greece | no | H1N1 infection or bacterial sepsis | not ARDS population |
| M. Schiller[5] | 2020 | cohort | Germany | no | not specified | not ARDS population |
| P. Sinha[6] | 2020 | prospective, observational | United Kingdom | yes | - | no non-COVID comparison |
| X. Thang[7] | 2021 | - | - | - | - | wrong DOI |
| B. Yu[8] | 2020 | retrospective | China | yes | community-acquired bacterial pneumonia | not ARDS population |
| J. Zhang[9] | 2021 | cohort | China | yes | seasonal influenza | not ARDS population |

***ARDS -** Acute respiratory distress syndrome

**Supplemental Figure 1 – Forest plot of D-dimer levels in subgroups**

**
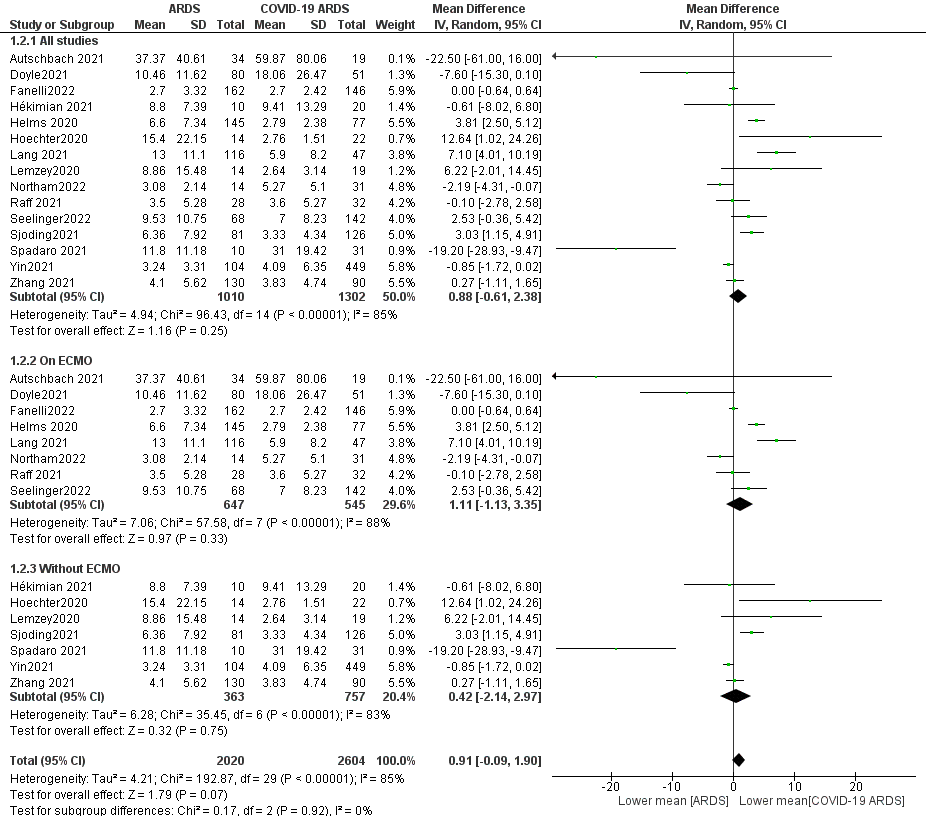
**

on ECMO: Patients in both groups were on ECMO from the start of the investigation

without ECMO: Patients in both groups were not on ECMO from the start of the investigation, but in some patient later got ECMO treatment because of the progression of their condition.

**Supplemental Figure 2 –** **Forest plot of CRP levels**

**
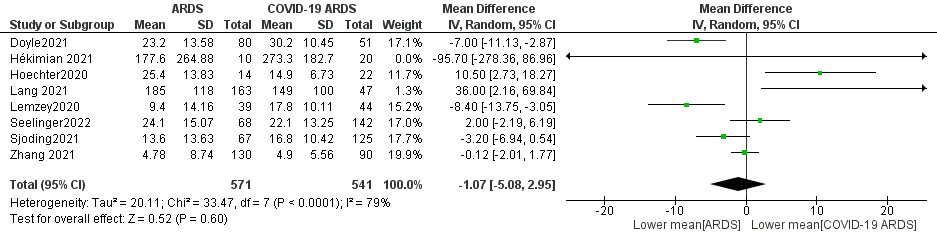
**

**Supplemental Figure 3 –** **Forest plot of fibrinogen levels**

**
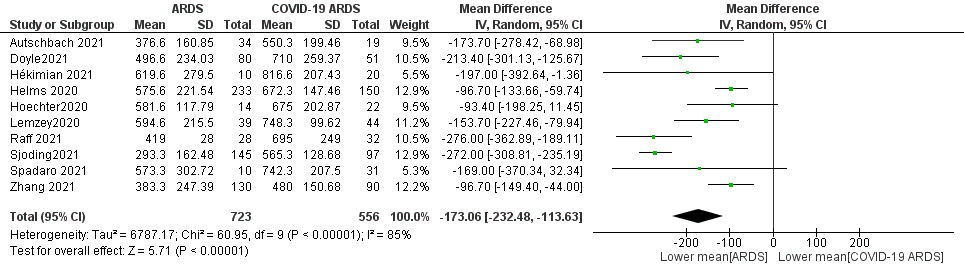
**

**Supplemental Figure 4 –** **Forest plot of PaO2/FiO2 ratio**

**
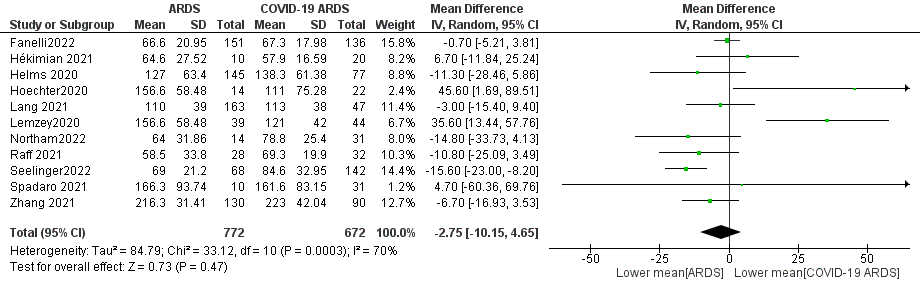
**

**Supplemental Figure 5 –** **Forest plot of length of Intensive Care Unit stay**

**
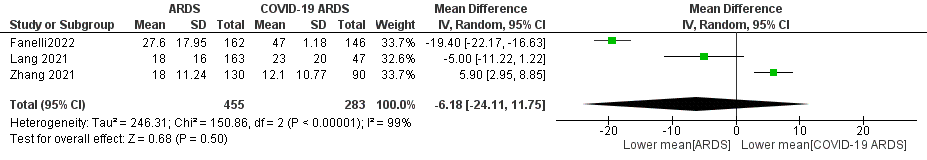
**

**Supplemental Figure 6 –** **Forest plot of length of hospital stay**

**
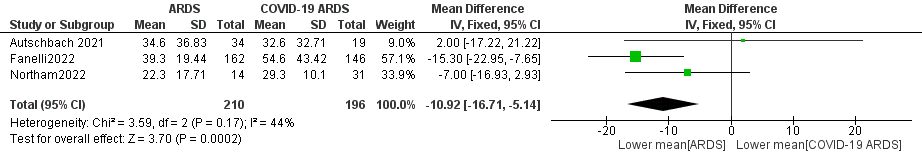
**

**Supplemental Figure 7 –** **Forest plot of mortality data at the longest follow-up available**

**
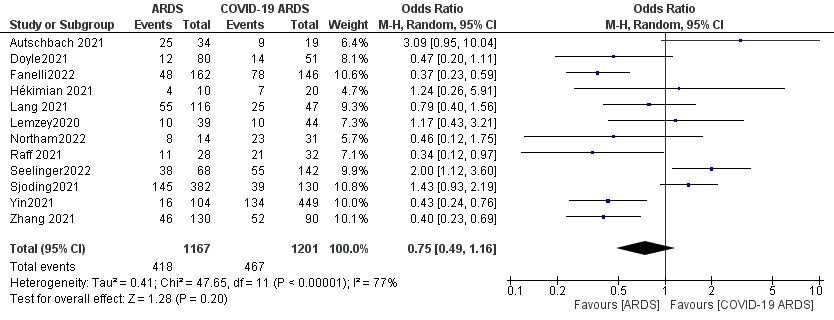
**

**Supplemental Figure 8 – Forest plot of ECMO duration**

**
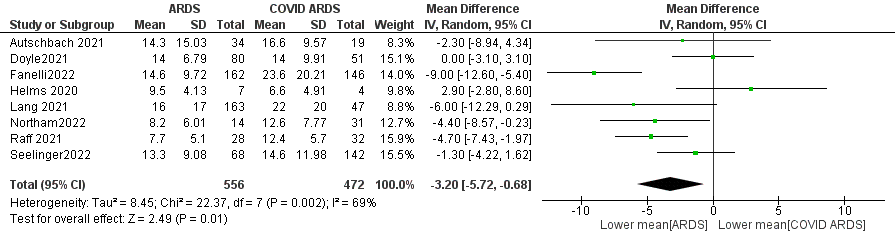
**

**Supplemental Figure 9 – Forest plot of D-dimer levels (sensitive analysis)**

**
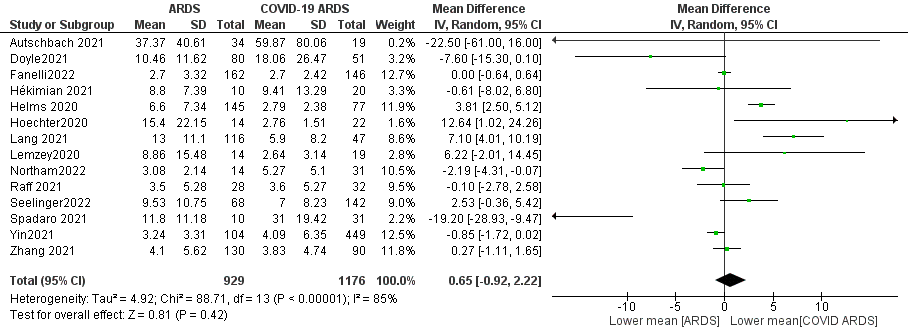
**

Excluded <7 points on the NOS scale.

**Supplemental Figure 10 –** **Funnel plot of D-dimer levels**

**
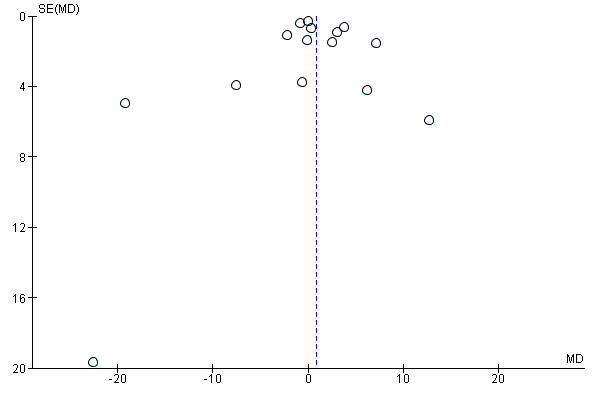
**

**Supplemental Figure 11 - Forest plot of D-dimer levels (removed unclear units)**

**
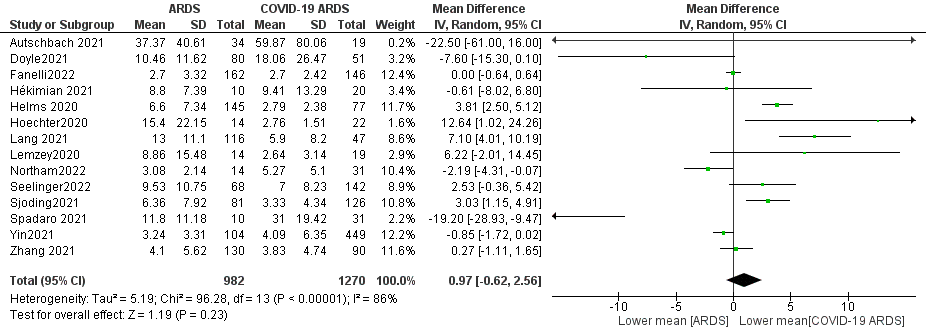
**

**Supplemental Table 3 - Thrombotic events (non-COVID ARDS vs. COVID ARDS) n/N”**

| **First author** | **D-dimer data collection time** | **Thrombotic events (non-COVID ARDS vs. COVID ARDS) n/N** |
| --- | --- | --- |
| T. Autschbach [26] | before ECMO | 4/34 vs.8/19 |
| A. J. Doyle [15] | before ECMO | Pulmonary immunothrombosis (4/80 vs 14/51) Deep vein thrombosis (15/80 vs.20/51) |
| V. Fanelli [17] | before ECMO | Cannula thrombosis (4/162 vs. 6/146) |
| G. Hékimian [18] | before ECMO | Pulmonary embolism (1/10 vs 2/20) |
| J. Helms [19] | at ICU admission | 9/145 vs.7/77 |
| D.J. Hoechter [20] | within 48 hours from ICU admission | no data available |
| C. N. Lang [22] | at admission | Pulmonary embolism (9/116 vs 10/47) stroke (embolic) (12/116 vs.2/47) |
| M. Lemzey [21] | at ICU admission | no data available |
| K. A. Northam [24] | during ECMO | 3/14 vs. 3/31 |
| L. A. Raff [12] | before ECMO | no data available |
| B. Seelinger [23] | before ECMO | 4/68 vs. 30/142 |
| M. W.Sjoding [14] | within 48 hours from intubation | no data available |
| S.Spadaro [25] | within 48 hours from start of mechanical ventilation | no data available |
| S. Yin [16] | at the time the patients met the definition of severe illness | no data available |
| J. Zhang [13] | at ICU admission | no data available |

**SUPPLEMENTAL REFERENCES**

1. Stang, A., *Critical evaluation of the Newcastle-Ottawa scale for the assessment of the quality of nonrandomized studies in meta-analyses.* Eur J Epidemiol, 2010. **25**(9): p. 603-5.

2. Grasselli, G., et al., *Pathophysiology of COVID-19-associated acute respiratory distress syndrome: a multicentre prospective observational study.* Lancet Respir Med, 2020. **8**(12): p. 1201-1208.

3. Mei, Y., et al., *Risk stratification of hospitalized COVID-19 patients through comparative studies of laboratory results with influenza.* EClinicalMedicine, 2020. **26**: p. 100475.

4. Papadakis, D.D., et al., *Immunostimulation and Coagulopathy in COVID-19 Compared to Patients With H1N1 Pneumonia or Bacterial Sepsis.* In Vivo, 2022. **36**(2): p. 954-960.

5. Schiller, M., et al., *Coronavirus disease (COVID-19): observations and lessons from primary medical care at a German community hospital.* J Community Hosp Intern Med Perspect, 2020. **10**(2): p. 81-87.

6. Sinha, P., et al., *Prevalence of phenotypes of acute respiratory distress syndrome in critically ill patients with COVID-19: a prospective observational study.* Lancet Respir Med, 2020. **8**(12): p. 1209-1218.

7. Tang, X., et al., *[Comparison of extracorporeal membrane oxygenation applicated in critical patients with COVID-19 and novel influenza A (H1N1) virus pneumonia].* Zhonghua Yi Xue Za Zhi, 2021. **101**(8): p. 579-585.

8. Yu, B., et al., *Evaluation of variation in D-dimer levels among COVID-19 and bacterial pneumonia: a retrospective analysis.* J Thromb Thrombolysis, 2020. **50**(3): p. 548-557.

9. Zhang, J., et al., *Platelet-driven coagulopathy in COVID-19 patients: in comparison to seasonal influenza cases.* Exp Hematol Oncol, 2021. **10**(1): p. 34.
